# Diffractive light-trapping transparent electrodes using zero-order suppression

**DOI:** 10.1515/nanoph-2023-0205

**Published:** 2023-08-03

**Authors:** Mengdi Sun, Di Huang, Pooria Golvari, Stephen M. Kuebler, Peter J. Delfyett, Pieter G. Kik

**Affiliations:** Bradley Department of Electrical and Computer Engineering, Virginia Tech, Arlington, 22203, VA, USA; CREOL, The College of Optics and Photonics, University of Central Florida, Orlando, 32816, FL, USA; Chemistry Department, University of Central Florida, Orlando, 32816, FL, USA; Department of Material Science and Engineering, University of Central Florida, Orlando, 32816, FL, USA; Physics Department, University of Central Florida, Orlando, 32816, FL, USA; Department of Electrical and Computer Engineering, University of Central Florida, Orlando, 32816, FL, USA

**Keywords:** binary diffractive gratings, light trapping, nanofabrication, transparent electrodes

## Abstract

A light-trapping transparent electrode design based on sub-surface binary dielectric gratings is introduced and demonstrated experimentally. The structure consists of metallic wires patterned with an array of silicon nanobeams. Optimization of the grating geometry achieves selective suppression of zero-order diffraction, while enabling redirection of incident light to an angle that exceeds critical angle of the local environment. Subsequent total-internal reflection allows recovery of light initially incident on the patterned metal wire. Experiments involving amorphous silicon gratings patterned on gold wires demonstrate a light-trapping efficiency exceeding 41 %. Modeling of crystalline silicon nanobeams on silver wires suggests that a shadowing loss reduction of 82 % is feasible. The achievement of a large shadowing reduction using a coplanar structure with high manufacturing tolerance and a polarization-insensitive optical response makes this design a promising candidate for integration in a wide range of real-world photonic devices.

## 1 Introduction

The trade-off between high optical transparency and high electrical conductivity is a long-standing challenge in many optoelectronic devices. In metallic wire-based electrodes, the optical transmission loss takes the form of shadowing, caused by reflection and absorption by the wire surface [1, 2]. To minimize shadowing losses, various approaches have been studied, including the use of solution-deposited metal nanowire networks [3–7], ultrathin metal films [8–11], regular metal wire array [12, 13], high aspect ratio metallic wires with small optical cross-section [14], triangular high-aspect ratio metallic wires that promote forward scattering [15, 16] and structures that direct light around metallic electrodes [17–21]. An intriguing approach to mitigating shadowing involves the trapping of light incident on metallic regions. A recent design utilized encapsulated metallic wires with inclined surface facets for redirection of incident light to angles beyond the critical angle of the encapsulation layer, leading to total internal reflection (TIR) and enabling near-complete elimination of shadowing losses [22–24]. This approach was recently demonstrated experimentally, exhibiting broadband polarization-independent suppression of shadowing [25]. Despite the high light trapping efficiency and high conductivity of such structures, the need for non-coplanar surfaces poses significant fabrication challenges. Here we demonstrate an approach that allows efficient light trapping using a co-planar design, based on judiciously designed nanoscale dielectric gratings defined on the surface of metallic wires.

**Figure 1:**
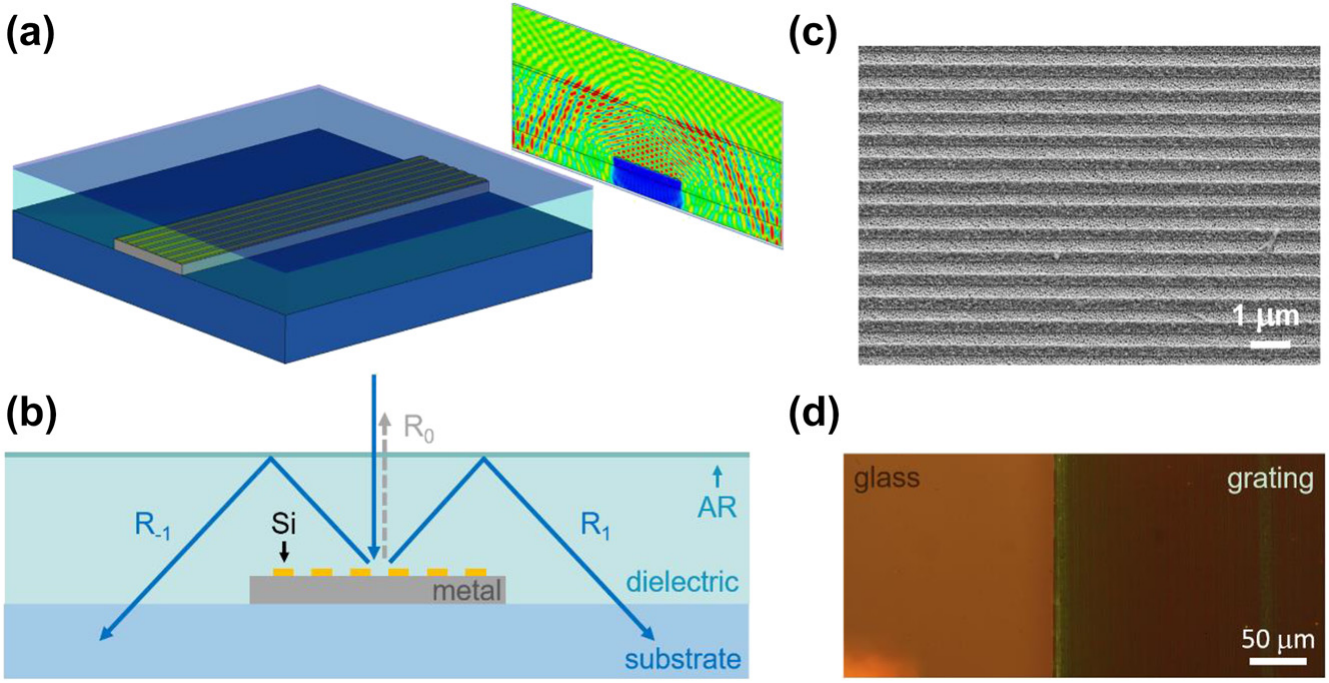
The schematics and the SEM/optical microscopy images of the diffractive light-trapping transparent electrodes. (a) Isolated diffractive light-trapping transparent electrode with a representative simulated electric field magnitude distribution. (b) Schematic electrode cross section showing the basic principle of operation. (c) SEM image of a Si grating on Au and (d) bright-field reflection microscopy image of the edge of a fabricated Si-on-Au light trapping diffractive electrode.

Figure 1(a) and (b) show the device under consideration. Light incident on a highly conductive metallic wire embedded in a transparent cover layer is diffracted by a dielectric grating defined on the wire surface. For sufficiently large diffraction angle the light will undergo TIR, redirecting the light into the substrate. This light trapping effect can dramatically reduce shadowing losses, provided specular reflection (diffraction into the 0th order) is suppressed. In the present study we demonstrate that destructive interference between reflection contributions from the top of the grating and the grating-metal interface can be used to suppress the zero order, while allowing efficient first-order diffraction followed by light trapping. One might conclude that light which would normally be diffracted into the zeroth order must therefore be absorbed by the structure. This however is not the case, because most of the incident optical power leaves the grating region by coupling into the +/−1 diffracted orders, after which it is redirected into the substrate *via* TIR. Figure 1(c) shows a scanning electron microscopy (SEM) image of an as-fabricated Si-based light trapping electrode, and Figure 1(d) shows a bright field reflection microscopy image of the same grating (right side). The grating-covered metallic region (right side) appears largely colorless and dark, indicating that reflection was successfully reduced across a wide spectral range that includes the entire visible spectrum. In the present study we numerically and experimentally investigate the performance of such diffractive light-trapping transparent electrodes.

## 2 Numerical analysis

**Figure 2:**
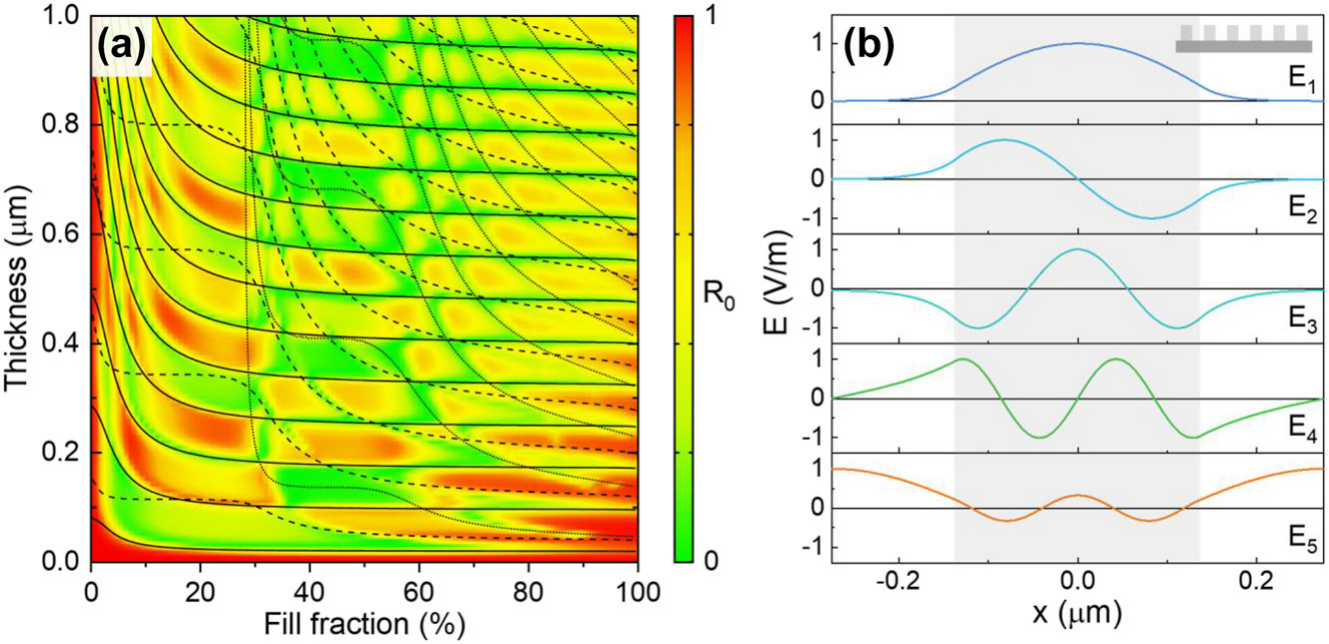
The simulation results from the RCWA and the modal analysis. (a) Normal-incidence zero-order reflection of a SiO_2_-embedded Si grating on silver as a function of Si areal fill fraction *f* and grating thickness for TE illumination at *λ*_0_ = 600 nm. Lines represent predicted locations of reflection minima based on a modal analysis, with minima from the first three excited grating modes shown as solid, dashed, and dotted lines, respectively. (b) Grating modes at *λ*_0_ = 600 nm for *f* = 50 % shown over a single grating period.

We first numerically study an extended binary silicon grating on a silver substrate embedded in a silica background. We consider the grating period *L*, the lateral Si fill fraction *f* (i.e., the duty cycle of the grating), and the grating thickness *D*. We initially select a wavelength of *λ*_0_ = 600 nm and a grating spacing of *L* = 550 nm, ensuring that the diffraction angle exceeds the critical angle of SiO_2_. The effect of the fill fraction *f* and the grating thickness *D* on the normal incidence reflection was studied with rigorous coupled wave analysis (RCWA) [26] using literature dielectric functions for Si, Ag, and SiO_2_ [27–29]. Figure 2(a) shows a contour graph of the power fraction reflected into the 0th diffraction order (*R*_0_) under TE illumination at *λ*_0_ = 600 nm. A complex pattern of reflection maxima and minima is observed. To understand these features, a model was developed that considers the pathways through which light can contribute to the zero-order reflection. The structure is divided into three regions: a homogeneous SiO_2_ cover layer, the patterned binary Si/SiO_2_ grating region, and a metal substrate representing a metallic wire. The incident wave is partly reflected into the 0th order at the top of the grating region, partly diffracted upward into the SiO_2_ region, and partly transmitted into distinct grating modes matching the periodicity of the grating. Symmetry prevents the excitation of anti-symmetric grating modes under normal incidence illumination. Any excited modes are reflected at the grating-metal interface and partially transmitted back into the zero-order or into the first diffracted order. We expect reduced reflection when the zero-order reflection from the grating surface destructively interferes with the wave reflected from the grating-metal interface, which occurs for the condition *D* = (*p* + ½) π *kzi* − 1 where *p* is an integer and *kzi* is the surface-normal wavevector of grating mode *i*. Under this condition, optical power is selectively directed to higher diffracted orders, while the zero-order diffraction (specular back-reflection) is suppressed. Figure 2(b) shows the five TE modes that match the periodicity of the grating for *f* = 50 %, obtained using the method described in Ref. [30]. The light gray shading indicates the location of the Si region of one grating period. The lowest order mode (*E*_1_, symmetric) resembles a waveguide mode in the Si region. The predicted location of reflection minima of this type of mode is indicated by the solid lines in Figure 2(a). Note that these curves closely follow the minima (green regions) in the simulated reflectance. The second excited mode (*E*_3_, symmetric) has a lower propagation constant, requiring a large grating thickness *D* to generate reflection minima (see the dashed lines in Figure 2(a)). Predicted reflection minima associated with the third excited mode (*E*_5_, symmetric) are shown with dotted lines. The good correspondence between the locations of the reflection minima calculated using RCWA (green regions) and the model predictions indicate that the observed zero-order suppression can indeed be understood in terms of destructive interference by two reflection contributions, one with a mode-dependent phase delay. Note that the minimum thickness for achieving a reflection minimum (lowest black solid line) is insensitive to the Si fill fraction since even at relatively low fill fractions the lowest order mode remains predominantly confined in the Si region, resulting in a consistently high propagation constant. As a result, Si-based diffractive light-trapping transparent electrodes are expected to be tolerant to patterning imperfections. Note that Figure 2 shows results for TE polarized illumination. Similar trends were observed for TM polarized illumination (see Figure S4 in the Supporting Information).

**Figure 3:**
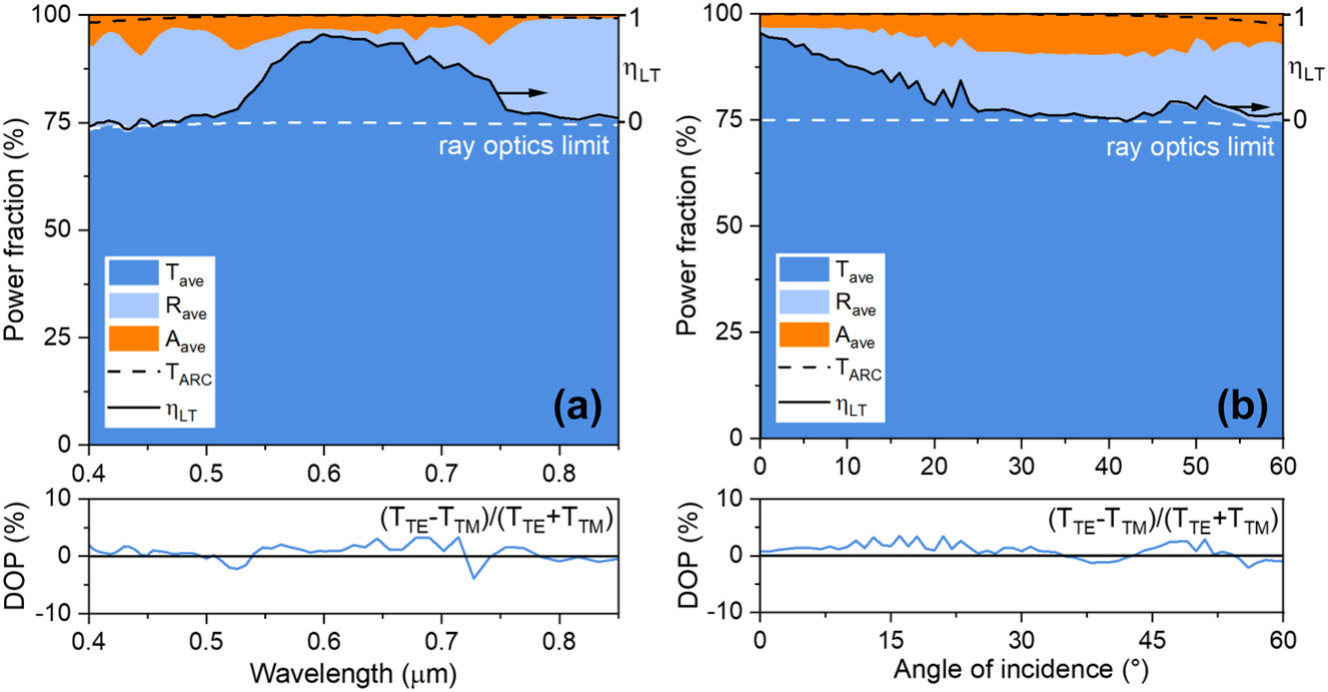
The simulation results from full wave electromagnetic analysis. (a) The spectral and (b) angular polarization-averaged transmission response of a diffractive light-trapping transparent electrode with a metal areal coverage of 25 % (top panel) and the corresponding degree of polarization of the transmitted light (bottom panel).

Next, we numerically evaluate the performance of an interdigitated diffractive light-trapping transparent electrode with parameters *L* = 550 nm, *D* = 30 nm, and *f* = 54 % (smallest Si feature size 300 nm) corresponding to a reflection minimum of *R* = 1 % in Figure 2(a). The periodically placed metal electrode lines have a width of *W* = 3850 nm as shown in Figure 1(a) and a metal areal coverage of 25 % (75 % open area). An anti-reflective coating (ARC) optimized for *λ*_0_ = 600 nm is placed on the silica cover layer. Since SiO_2_ has a refractive index of ∼1.5 at *λ*_0_ = 600 nm, the first order diffraction angle is *θ* = 46.7°, which is larger than the critical angle (41.8° for a SiO_2_/Air interface), enabling TIR-based light trapping. To minimize the risk of secondary shadowing [24] in which recovered light hits the original electrode or an adjacent electrode, the cover layer thickness is set to 2310 nm. In our design, the sheet resistance of the metal wire array is given by *R*_sh_ = *ρ*/*t*_eff_, where *ρ* is the resistivity of the material and *t*_eff_ = *t*·*f* is the surface-averaged metal thickness, with *t* the metal wire thickness and *f* the metal areal coverage. The resistivity of silver is 1.6 × 10^−8^ Ω m and the metal thickness is set to 400 nm. Given *f* = 25 %, the corresponding sheet resistance is 0.16 Ω/sq. The response of the diffractive trapping electrode design was evaluated using finite element based simulation [31]. In Figure 3(a) we investigated the wavelength dependent transmission, reflection, and absorption of the structure in Figure 1 for unpolarized illumination (*T*_ave_), representative of unpolarized solar irradiation, as well as the degree of polarization DOP = (*T*_TE_ − *T*_TM_)/(*T*_TE_ + *T*_TM_). To better visualize the relative variation between different power fractions of the incident light, here the polarization-averaged transmission (*T*_ave_), reflection (*R*_ave_) and absorption (*A*_ave_) are represented by dark blue, light blue and orange color bars, respectively. The ray optics response with metal wires but without the grating (no light trapping, white dashed line) and without patterned metal wires (no shading losses, black dashed line) are included. The latter curve, which we call *T*_max_ represents the transmission under perfect trapping of light incident on the metallized regions. Figure 3(a) also includes the light-trapping efficiency (black solid line) given by *η*_LT_ = (*T* − *T*_min_)/(*T*_max_ − *T*_min_) where *T*_min_ = (1 − *f*)*T*_max_ represents the ray-optics prediction of the transmission in the absence of the grating. Defined this way, *η*_LT_ represents the shadowing reduction, with *η*_LT_ = 100 % corresponding to the complete elimination of shadowing. A light trapping efficiency of 82.3 % is observed at a wavelength of 600 nm, corresponding to a more than five-fold reduction in shadowing. This result is quite close to the best-case prediction of 92.6 % based on RCWA calculations for an infinitely extended grating. Light trapping efficiencies above 30 % are observed in the wavelength range 540 nm–740 nm, with a spectrally averaged transmission of *T* = 91 % and an average light-trapping efficiency of *η*_LT_ = 62 % for unpolarized light. A relatively rapid efficiency drop occurs for short and long wavelengths, predominantly due to wavelength-dependent diffraction. At *λ*_0_ > 750 nm, diffracted light can reach the neighboring electrode line where it may partly be redirected out of the structure, an effect called secondary shadowing [24]. At *λ*_0_ > 800 nm, no diffracted orders can be generated in the cover layer, preventing diffraction-based light trapping. At wavelengths shorter than 550 nm, the diffracted angles fall below the critical angle, resulting in a loss of TIR and low light trapping efficiency. Figure 3(b) shows the same quantities for *λ*_0_ = 600 nm as a function of incident angle. As the angle of incidence increases, the light trapping efficiency drops due in part to the loss of TIR for one of the diffracted orders as the incident angle changes. Despite these effects, a transmission of *T* > 82 % is achieved with *η*_LT_ > 30 % over a 36° angular range about the surface normal. The transmission is relatively polarization independent, with a degree of polarization below 4 % for all angles and wavelengths.

## 3 Experimental validation

**Figure 4:**
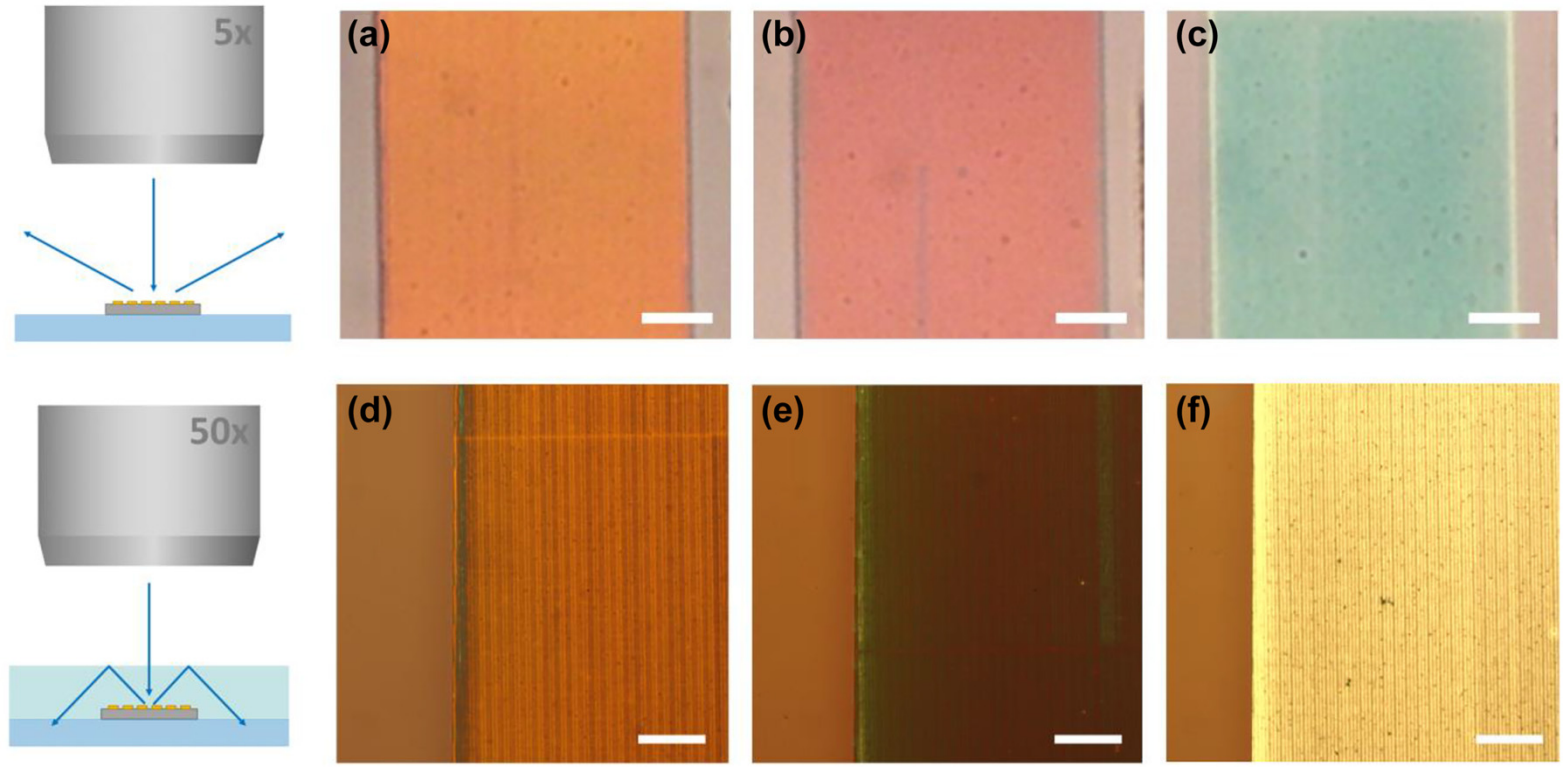
Bright-field reflection microscopy images of diffractive light trapping structures with silicon fill fractions *f* = 30 %, 54 %, and 80 % (a)–(c) at 5× magnification (uncovered); and (d)–(f) at 50× magnification in the light-trapping geometry (cover slip and index matching oil applied). The scale bars represent 100 μm and 50 μm, respectively.

Proof-of-concept light trapping electrodes were fabricated using e-beam lithography on Au wire regions, followed by Si deposition, dry etching, and lift-off (see Supplementary material). Figure 4 shows brightfield reflection microscopy images of the samples with and without a light-trapping cover layer, and corresponding measurement schematics. Here we use Au instead of Ag to fabricate the metallic wires since gold is less susceptible to oxidation and corrosion compared to Ag films. Since the optical response of thick gold and silver films is almost identical in the visible spectrum (close to 100 % back-reflection), gold wires are an appropriate choice for a proof-of-concept demonstration. Figure 4(a–c) show bright-field reflection images of a 0.48 mm wide Au wire on ITO coated glass, covered with binary Si gratings with a grating period of *L* = 550 nm and Si areal fill fractions *f* = 30 %, 54 % and 80 %, taken using a 5× objective with N.A. = 0.15, corresponding to a maximum collection angle of 9°. A small aperture stop was used to ensure near-normal illumination. The different gratings produce distinct colors, suggesting a substantially different zero-order reflection spectrum despite having the same grating period. Next, the structures were embedded in index-matching liquid (*n* = 1.52), and a cover slip was placed on top such that the two form a nearly uniform dielectric cover layer, representing a light-trapping geometry. In the case of efficient broadband light-trapping we expect the grating to appear dark, showing no pronounced color. Figure 4(d) shows a structure with Si fill fraction of *f* = 30 %, taken near the left edge of the grating region using a 50× objective (N.A. = 0.8). Note that the grating-covered gold wire has a similar brightness as the glass region (left side of image), suggesting remarkably low reflection from the metallic region. The grating region has a reddish hue, indicating that short wavelength light is either trapped or absorbed by the grating region. Figure 4(e) shows the region with a Si fill fraction of *f* = 54 %. Note that the metallic region appears darker than the surrounding glass region, indicating remarkably efficient zero-order suppression and a potentially larger light trapping efficiency than the *f* = 30 % region. Finally, Figure 4(f) shows the region with a Si fill fraction of *f* = 80 %. This region appears significantly brighter than the grating regions in Figure 4(c) and (d), indicating inefficient zero-order suppression.

**Figure 5:**
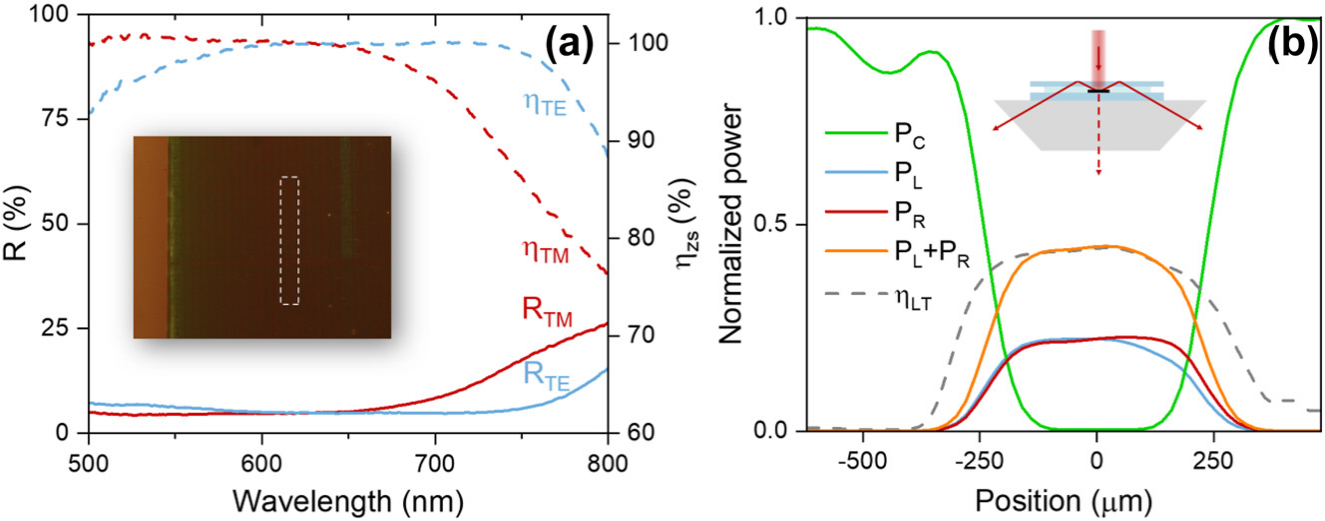
The spectral responses and the results of the linear laser scanning of the electrodes sample. (a) Reflection spectrum (solid lines) and corresponding zero-order suppression efficiency (dashed dot lines) for TE and TM polarizations. (b) Spatial dependence of recovered power of on-axis (*P*_*C*_) and off-axis (*P*_*L*_ and *P*_*R*_) transmission under TM polarized illumination.

To assess the spectral dependence of the zero-order suppression, we measured the reflection spectrum (*R*) of the *f* = 54 % electrodes at different polarizations using a microscope-coupled spectrometer. A 5× objective (N.A. = 0.15) was used, and a small aperture stop was placed in the illumination path. The measured reflection spectra for TE and TM polarized light are shown in Figure 5(a) as blue and red lines respectively. The collection area is shown schematically by the dashed rectangle in the inset. Low reflectance (<8 %) is observed from 500 nm–700 nm for both polarizations. The results suggest that zero-order suppression is effective across a wide spectral range, matching the predictions made in Figure 3(a). Figure 5 shows the zero-order suppression efficiency, defined as *η*_*zs*_ = 1 − (*R*_*G*_ − *R*_*C*_)/(*R*_*M*_ − *R*_*C*_), in which *R*_*G*_, *R*_*M*_ and *R*_*C*_ are the measured reflectance values from the grating region, a glass-covered bare Au metal stripe, and the surface of an isolated cover slip (Figure 5(a), dashed lines), respectively. For both TE and TM polarization, near-complete zero-order suppression is observed in the range 600 nm–650 nm, following the simulation results in Figure 3(a).

The high zero-order suppression efficiencies observed in Figure 5(a) are attributed to a combination of light trapping and absorption by the a-Si and the underlying gold film. To find the relative contributions of these factors, we measure the fraction of trapped light under illumination of the electrode with *f* = 54 %. The sample is placed on a prism to allow measurement of off-axis recovered (trapped) light, and index matching oil is applied between sample and prism. The measurement geometry is shown schematically in the inset of Figure 5(b). A HeNe laser (*λ*_0_ = 633 nm) with a FWHM spot size of ∼300 μm was used as the illumination source. The sample position was scanned such that the grating-coated wire crossed the laser beam. Optical power meters were placed on-axis and off-axis at an angle close to 50°. The power readings were used to determine the light trapping efficiency *η*_LT_ = (*P*_*L*_ + *P*_*R*_)/(*P*_*C*,max_ − *P*_*C*,min_), in which *P*_*L*_ (*P*_*R*_) is the off-axis power collected on the left (right) side, corresponding to the trapped −1 (+1) diffracted order, and *P*_*C*,min_ and *P*_*C*,max_ are the on-axis power when the laser is incident on the grating region and on a transparent area near the light trapping electrode, respectively. Figure 5(b) shows the position-dependent on-axis power (*P*_*C*_) and the off-axis recovered power (*P*_*L*_ + *P*_*R*_) under TM-polarized illumination as the incident laser spot is scanned across the grating with a step size of 20 μm. As the laser spot moves across the patterned wire, the on-axis signal (green line) reaches near-zero transmission (<1 %), which would correspond to strong shadowing by the metallic wire in the absence of light trapping. However, as the on-axis signal drops, the off-axis recovered power by both the left and right detectors (blue and red lines) gradually increases from zero to ∼22 % when the laser spot is near the center of the electrode, indicating up to 44 % of the light incident on the opaque electrode line is recovered by the grating. The corresponding result under TE-polarized illumination is shown in Figure S2 of the Supporting Information, showing similar trends in on-axis and off-axis transmission. TE polarized illumination resulted in a slightly lower peak light-trapping efficiency of ∼38 %, resulting in a polarization-averaged light trapping efficiency of 41 %. These losses, attributed to absorption in the amorphous Si grating and in the gold film, may be mitigated by switching to lower loss dielectrics and metals. While such material optimization is outside the scope of the present proof-of-concept demonstration, these aspects could be investigated in future experimental studies. Comparing these light trapping efficiencies with the near-perfect zero-order suppression efficiency at 633 nm (Figure 5(a)), as much as 56 % of the incident light may be absorbed by the light trapping grating under TM-polarized illumination. Given the measured light trapping efficiency and the resistivity of Au [32], an Au electrode with 10 % areal coverage would exhibit an optical transmission of *T* = 94 % and an effective sheet resistance of *R*_sh_ = 0.73 Ω/sq, which compares favorably to the state-of-the-art light trapping electrodes. For example, transparent electrodes based on silver nanowire network reported by van de Groep et al. [13] achieve 91 % transmission at 6.5 Ω/sq sheet resistance, which shows relatively lower conductivity due to limited thickness of the wires. Cloaked contact grids reported by Schumann et al. [18] have a working wavelength of 1550 nm due to the resolution of direct laser writing, which limits its application in the visible range. In addition, the co-planar nature of the electrode presented here and the use of dielectric materials make these structures compatible with fabrication techniques such as electron beam lithography and nano-imprint lithography, offering significant advantages in manufacturability compared to non-coplanar light trapping geometries. As a result, our design can be fabricated on arbitrarily large electrodes, enabling more efficient charge carrier extraction.

## 4 Conclusions

In summary, light-trapping transparent electrodes consisting of binary embedded dielectric gratings patterned on metallic wires were theoretically and experimentally investigated. Shadowing losses in an electrode with a 25 % metal coverage were numerically predicted to be reduced by more than a factor five under normal incidence illumination at 600 nm, and the simulated light trapping efficiency was found to exceed 30 % in the wavelength range 540 nm–740 nm. The light trapping mechanism was found to be relatively insensitive to polarization. Proof-of-concept diffractive light trapping electrodes showed a polarization-averaged light trapping efficiency of 41 % for unpolarized illumination at normal incidence at a wavelength of 633 nm. Additionally, the co-planar and the dielectric nature make our design compatible with standard semiconductor fabrication techniques such as electron beam lithography and nano-imprint lithography, showing greater potential in industrial manufacturing over non-coplanar light trapping electrodes. Due to the intricate nature of the presented design, initial implementations might involve advanced devices where maximum performance is critical, even if that incurs high fabrication costs, for example in advanced high-speed photodetectors and other advanced optoelectronic devices.

## 5 Methods

### 5.1 Sample fabrication

Proof-of-concept samples were fabricated on an ITO coated glass substrate on a multi-step basis. Detailed information of the process is included in the Supplementary material.

### 5.2 Optical characterization

Optical microscopy images were taken in an Olympus BX51TRF microscope in bright-field reflection mode under the illumination of unpolarized white light. Images of a light-trapping samples were taken by placing index matching oil (Cargille non-drying immersion oil for microscopy, *n* = 1.518) between the sample and a #1.5 cover slip (thickness 170 µm). Spatially resolved transmission measurements were carried out using a 633 nm He–Ne laser. The prototype electrodes were illuminated with both TE and TM polarized light (electric field parallel/perpendicular to the long axis of the wire). The transmitted power was measured using a Newport 918D-SL-OD3R silicon photodetector. The reflection spectrum was measured by a Horiba iHR 320 imaging spectrometer.

## Supplementary Material

The online version offers supplementary material on the details of the fabrication process of the prototype electrode samples, the spatial dependence of recovered power under TE polarized illumination, RCWA calculations of the zero-order diffraction of a Si-grating covered gold film under TE polarized illumination and RCWA calculations of the geometry-dependent zero-order diffraction under TM polarized illumination.

## Supplementary Material

Supplementary Material Details
