# Supplementary material for: Diffractive light-trapping transparent electrodes using zero-order suppression
Source: Nanophotonics. 2023 Aug 3;12(18):3545–52. doi: 10.1515/nanoph-2023-0205 (PMC11501630; doi:10.1515/nanoph-2023-0205)
Supplement: Supplementary file 1 — Supplementary Material Details [file j_nanoph-2023-0205_suppl_001.pdf]

Supplementary material: Diffractive light-trapping transparent electrodes using zero-order suppression

Mengdi Sun[1]*, Di Huang[2], Pooria Golvari[3], Stephen M. Kuebler[2,3,4], Peter J. Delfyett[2,5,6] and Pieter G. Kik[2,5]*

[1]Bradley Department of Electrical and Computer Engineering, Virginia Tech, Arlington, 22203, Virginia, USA

[2]CREOL, The College of Optics and Photonics, University of Central Florida, Orlando, 32816, Florida, USA

[3]Chemistry Department, University of Central Florida, Orlando, 32816, Florida, USA

[4]Department of Material Science and Engineering, University of Central Florida, Orlando, 32816, Florida, USA

[5]Physics Department, University of Central Florida, Orlando, 32816, Florida, USA

[6]Department of Electrical and Computer Engineering, University of Central Florida, Orlando, 32816, Florida, USA

Fabrication process

The prototype device was fabricated in a multi-step process. Figure S1 shows the sample fabrication steps. In brief, photolithography was performed in NR7-1000PY negative photoresist using MJB3 mask aligner to expose the ITO-coated glass substrate for further deposition of 480-µm wide gold strips. Titanium (5 nm) was deposited to improve adhesion of the ITO-coated glass substrate to gold, followed by the deposition of a 300-nm layer of gold (Edwards thermal evaporator). Amorphous silicon (32 nm) was deposited on top of the gold layer using temescal FC-2000 E-beam evaporator at 4×10^{-7} torr.

The zero-order suppression grating was fabricated on a gold stripe using the following procedures: a positive e-beam resist (495PMMA A6, MicroChem) was spin-coated on the gold-coated substrate at 3500 rpm (60 s) and pre-baked (180 °C, 1 min) to give a 300-nm film. The resist was patterned by e-beam lithography using Leica EBPG 5000+. An acceleration voltage of 50 keV, beam current of 10 nA, and exposure dose of 200 μC/cm^2 were used. The exposed substrate was immersed in a 3 : 7 mixture (v/v) of IPA : deionized water for 2 min to develop the grating patterns on the gold stripe. The grating patterns were then transferred into the Si layer by reactive ion etching (RIE) using a CF_4 : SF_6 (50 : 70) gas mixture. Finally, the substrate was immersed in acetone for 12 h to lift off the photolithography mask and remove the e-beam resist.

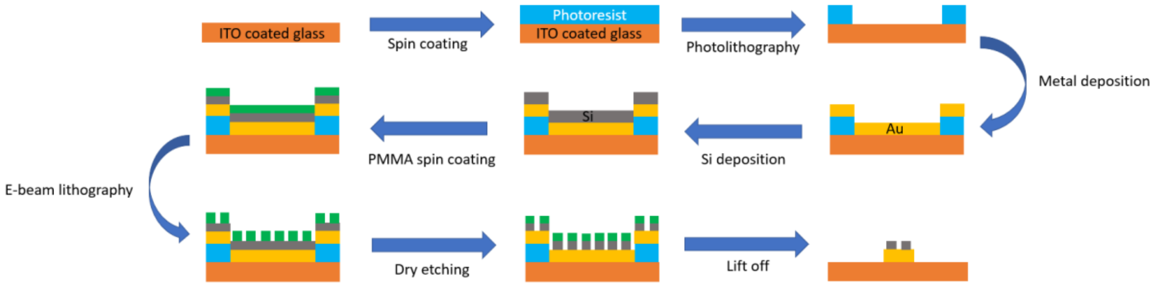

Figure S1. Process flow of the fabrication of grating-based light-trapping transparent electrodes.

Spatial dependence of transmitted and recovered power under TE polarized illumination

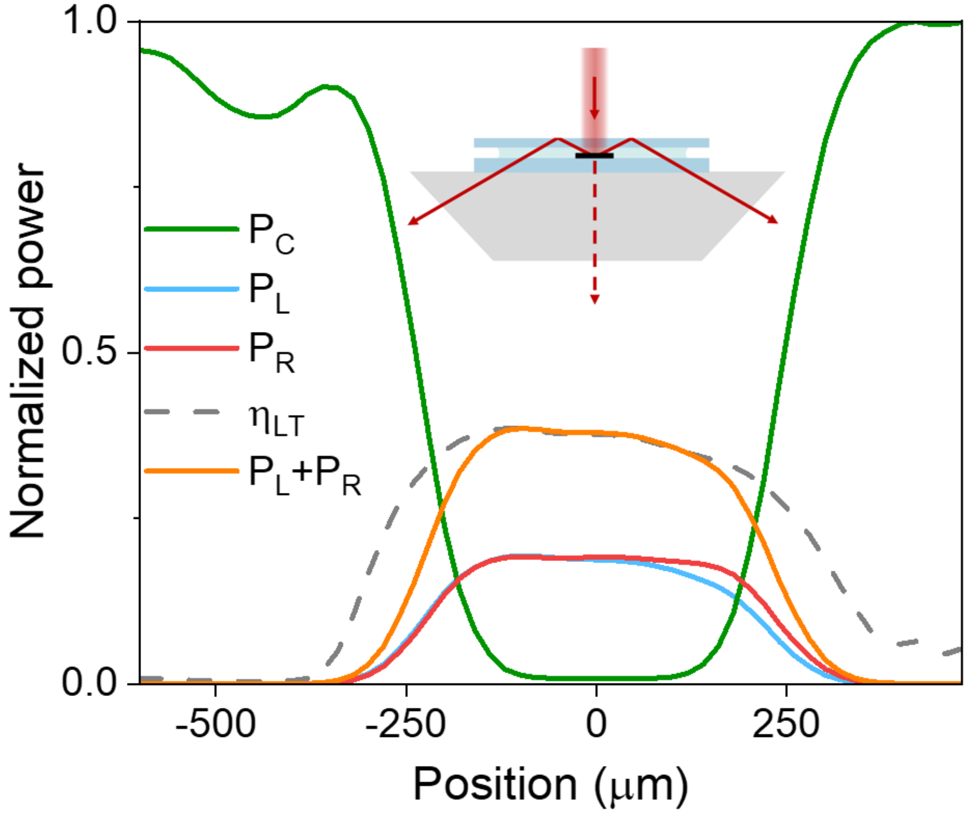

Figure S2. Spatial dependence of transmitted on-axis power (solid green line) and recovered off-axis power (solid blue and red lines) under TE polarized illumination.

RCWA calculations of the zero-order diffraction of a Si-grating covered gold film

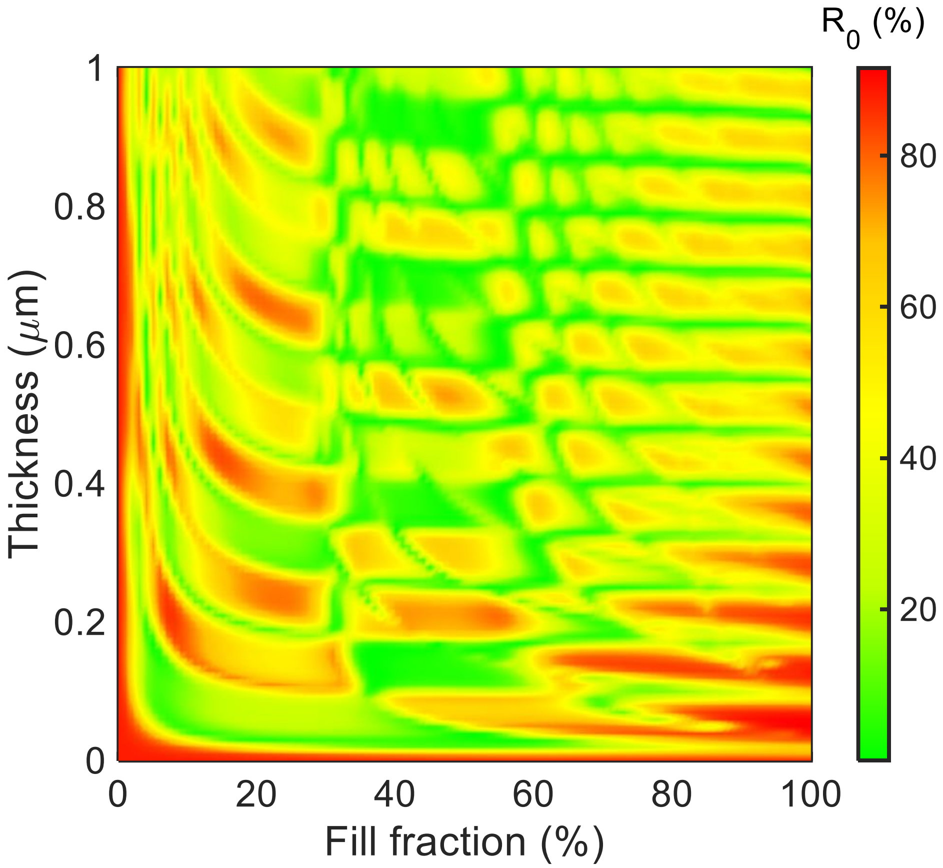

Figure S3. Normal-incidence zero-order diffraction of a SiO_2-embedded Si grating on a gold film as a function of Si areal fill fraction *f* and grating thickness for TE and illumination at λ_0 = 600 nm.

RCWA calculations of the zero-order diffraction of a Si-grating covered silver film under TM illumination

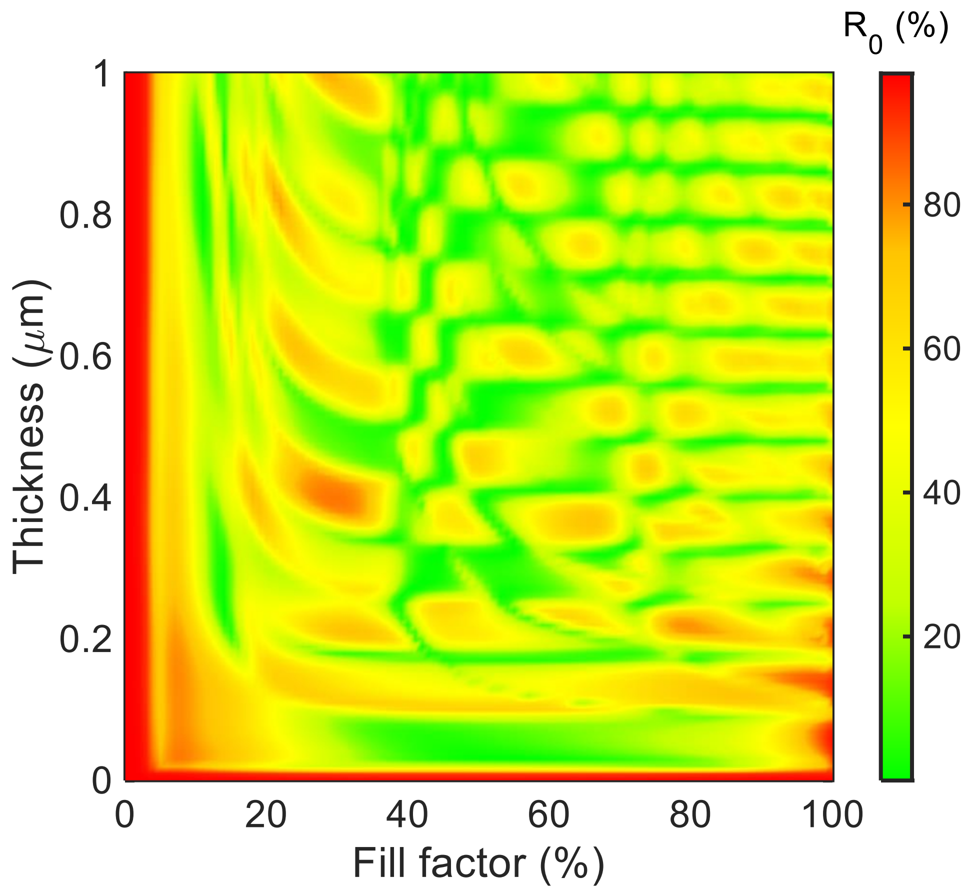

Figure S4. Normal-incidence zero-order diffraction of a SiO_2-embedded Si grating on silver as a function of Si areal fill fraction *f* and grating thickness for TM illumination at λ_0 = 600 nm.
